# COVID-19 and its prevention in internally displaced person (IDP) camps in Somalia: impact on livelihood, food security and mental health

**DOI:** 10.1186/s12889-022-14878-z

**Published:** 2022-12-22

**Authors:** Farah I. Mumin, Fred D. Wesonga, Jibril I. M. Handuleh, Ross G. White, Siobhan M. Mor

**Affiliations:** 1 Faculty of Veterinary Medicine, Red Sea University, Bosaso, Somalia; 2 IGAD Sheikh Technical Veterinary School, Sheikh , Somaliland; 3 Department of Psychiatry, Saint Paul’s Hospital Millennium Medical College, Addis Ababa, Ethiopia; 4 School of Public Health, Amoud University, Borama, Somaliland; 5 School of Psychology, Queen’s University Belfast, Belfast, UK; 6 Institute of Infection, Veterinary and Ecological Sciences, University of Liverpool, Liverpool, UK; 7 International Livestock Research Institute, Addis Ababa, Ethiopia

**Keywords:** COVID-19, Somalia, Internally displaced persons, Livelihood, Mental health

## Abstract

**Background:**

Somalia has over 2.6 million internally displaced people (IDP) that depend on daily wages and humanitarian assistance for their livelihoods. This study investigated the impact of COVID-19 on livelihoods, food security and mental health of Somalia’s IDPs.

**Methods:**

A questionnaire was conducted with “breadwinners” (*n* = 585) residing in 15 randomly selected IDP camps. Mental health was assessed using the 5-item World Health Organization Wellbeing Index (WHO-5) and the Patient Health Questionnaire-9 (PHQ-9). Multivariable regression was used to explore the effect of depressive symptoms on soap use and ability to pay for food/medicine/rent.

**Results:**

Knowledge of COVID-19 symptoms, transmission and prevention was relatively high, however only 55% reported using soap for hand washing. Around one third perceived that prohibition of public gatherings had negatively impacted weekly earnings. Participants reported difficulty buying food (85%), medicine (82%) and paying rent (51%) because of COVID-19. The majority were assessed as having low wellbeing and high depressive symptoms (mean WHO-5 = 44.2/100; mean PHQ-9 = 18.6/27), with most (74%) indicating that they felt worse than before the pandemic. Compared to people with low depressive symptoms, people with high depressive symptoms were less likely to use soap (aOR = 0.3, 95% CI = 0.2, 0.7; *P* < 0.001) and more likely to report difficulty buying food (aOR = 2.2; 95% CI = 1.1, 4.3; *P* = 0.02).

**Conclusion:**

COVID-19 and associated restrictions have negatively impacted Somalia’s internally displaced population. Livelihood and mental health support is urgently needed in the recovery phase of the pandemic and should be factored into future pandemic planning.

**Supplementary Information:**

The online version contains supplementary material available at 10.1186/s12889-022-14878-z.

## Introduction

COVID-19 preventive measures such as hand washing and social distancing are challenging to implement for approximately one billion people living in informal settlements or urban slums worldwide due to insufficiency or lack of basic needs such as water, waste management, and inadequate housing which increases vulnerability to infection [1–3]. The world’s forcibly displaced population (estimated at 89.3 million people in 2021, including 53.2 million internally displaced [4]) are amongst the most vulnerable of communities in terms of living standards [5] and they have a high burden of mental health disorders, especially when the reasons for displacement relate to conflict and political unrest [6–9]. This includes in Somalia, where decades of conflict and insecurity, as well as extreme climate events, have given rise to an estimated 2.9 million internally displaced people (IDP) [10, 11].

Somalia’s fragile health system makes the country particularly vulnerable to COVID-19. In 2019, Somalia ranked 193/195 in terms of its ability to respond to a pandemic according to the Global Health Security Index [12]. Except for a few public hospitals operating under donor support, most of the health providers are privately owned clinics and hospitals in the big cities which are either too expensive or too far from IDPs living in more urban neighborhoods. When the pandemic emerged, Somalia had no equipped reference laboratories for testing and few Intensive Care Unit beds and ventilators were available [13]. Subsequently the Ministry of Health established a multi-sectoral task force which issued a contingency plan for preparedness and response by following WHO preventive guidelines [14]. As part of this plan, isolation wards with limited capacity were established in dedicated hospitals in Mogadishu, Garowe and Hargeisa and temperature screening was initiated at the four major airports [15].

After the first case of COVID-19 was reported in Mogadishu (16 March 2021) and spread to federal member states, the government introduced restrictions on domestic and international travel, closed educational institutions and banned public gatherings [16]. Whilst these measures likely slowed the spread of COVID-19, they may have disproportionaly impacted the livelihood of IDPs, many of whom depend on humanitarian aid and/or make a living from daily undertaking by travelling to the city markets and seaports. As of 24th April 2022, there have been 26,485 cases and 1,361 deaths reported in Somalia [17]. However, given the limited diagnostic capacity in the country, these numbers likely underestimate the magnitude and impact of the disease in the population [18], particularly in disadvantaged, internally displaced communities [19].

Following the emergence and spread of SARS-CoV-2, the shelter cluster recognized 98,800 households living in 237 IDP camps across Somalia as having a high risk of virus transmission [20, 21]. In partnership with the local governments, local and international NGOs conducted COVID-19 awareness campaigns and distributed handwashing stations throughout the country which contributed to awareness of the pandemic and non-pharmaceutical preventive measures amongst IDP camp residents [22, 23]. Subsequently, we conducted a survey between December 2020 and March 2021 to assess the level of COVID-19 awareness, knowledge and attitudes; hand hygiene practices and attitudes; and perceived impacts of COVID-19 restrictions on livelihoods and food security of Somalia’s IDPs. Further, given the potential for COVID-19 and associated restrictions to exacerbate the high pre-existing burden of mental health disorders in these communities, we also assessed mental health and wellbeing impacts and its association with current soap use and ability to pay for rent and buy food and medicine. Whilst the pandemic is recognised as having remarkable mental health implications in many countries [24–27] including Somalia [28–31], no studies have been done to understand mental and livelihood impacts of COVID-19 to Somalia’s over 2.6 million internally displaced community.

## Methods

### Study areas

This study was undertaken in four purposively selected locations, namely Mogadishu, Hargeisa, Garowe and Baidao cities. These locations were selected because they had amongst the highest number of confirmed COVID-19 cases as of June 2020 (when the study protocol was developed); a large population of IDPs; and well-established camp management as supported by the Camp Coordination and Camp Management (CCCM) cluster of the International Organization of Migration (IOM) in Somalia. According to data provided by the CCCM cluster during the design phase of the project, there were a total of 165,351 households living in 1,631 IDP camps across the four selected cities of Somalia.

### Selection of settlements and participants

Based on the estimated number of IDP households in the selected cities (*n* = 165,351), and assuming 50% knowledge with an absolute precision of 5% and 95% confidence interval, a sample size of 384 was determined using Epitools (https://epitools.ausvet.com.au/oneproportion). Since multi-stage sampling was used, a design effect was applied to correct for clustering. Cluster surveys performed in humanitarian settings in Somalia and elsewhere have tended to use a design effect of between 1.2 and 2 [32]. Since no previous studies had investigated mental health and wellbeing impacts of COVID-19 in this setting, we applied a design effect in the middle of this range (i.e. 1.5), giving a minimum sample size of 576.

A list of all IDP settlements in the four selected cities was obtained from CCCM. Fifteen camps were randomly selected with a probability proportional to the size of the number of IDP households in each city. Thus, five settlements were randomly selected from Baidao, one from Garowe, three from Hargeisa and six from Mogadishu. A fixed number of households (*n* = 39) was subsequently sampled from each of the fifteen randomly drawn IDP settlements, for a final sample size of 585. Interviewers sought permission to visit camp residents from the nearest local government and camp management offices in each location by submitting a short written summary about the project with oral elaboration provided where needed. Where permission was not granted a different settlement was randomly selected (i.e. settlements were sampled with replacement). Male and female “breadwinners” (primary income earners in a household) who were aged 18 years or above and who were residents in selected IDP settlements were eligible to participate. Where possible a list of all camp residents in the selected settlement was obtained from the camp management to draw a sampling frame. Simple random sampling was employed to select individual households. Where this was not possible, transect walks were used to select the households. In this case, data collectors went to the center of the camp and spun a bottle/pen to determine the direction of the transect. Data collectors then walked in this direction and selected households using systematic random sampling (e.g. every 3rd household), with the sampling interval varying based on the size of the camp.

### Data collection

A detailed, semi-structured questionnaire was developed to gather relevant information related to COVID-19 awareness, knowledge and attitudes; hand hygiene practices and attitudes; and perceived impacts of COVID-19 restrictions on livelihoods, food security and mental health. Knowledge, awareness and attitudes questions were partly informed by a prior Risk Communication and Community Engagement assessment undertaken by CCCM Cluster partners [22]. Mental health and wellbeing was assessed using standardized tools that have been previously used in IDP/refugee settings [33, 34], namely the 5-item World Health Organization Wellbeing Index (WHO-5) and the Patient Health Questionnaire-9 (PHQ-9). Both tools were translated into Somali language by an experienced psychiatrist who also provided guidance for data collectors on the Somali terminologies to use during interviews. The WHO-5 is a widely used and validated measurement of current psychological wellbeing [35]. The positively phrased WHO-5 tool assesses wellbeing using the following statements: 1) ‘I have felt cheerful and in good spirits’, 2) ‘I have felt calm and relaxed’, 3) ‘I have felt active and vigorous’, 4) ‘I woke up feeling fresh and rested’ and (5) ‘My daily life has been filled with things that interest me’ [35]. Respondents were asked to consider their situation in the last 14 days and score these statements from 0 to 5 according to frequency (0 = at no time, 1 = some of the time, 2 = less than half of the time, 3 = more than half of the time, 4 = most of the time and 5 = all the time). A WHO-5 score less than < 50 was classified as a suggestive of possible depression [35]. The PHQ-9 assesses the presence of depressive symptoms by asking participants to indicate how frequently they experience symptoms such as “feeling down, depressed or hopeless” or “thoughts that you would be better off dead or of hurting yourself in some way”. For each question, respondents were asked to assign a score based on frequency (0 = nearly every day, 1 more than half the days, 2 = several days, 3 = not at all). Questionnaires were implemented using KoBo Toolbox (Harvard Humanitarian Initiative, Cambridge, MA), an open-source platform based on Open Data Kit (ODK).

Interviews were conducted by licensed public health professionals who were fluent in Somali language. A quiet place, separate from crowded areas was located with the help of each interviewee. A detailed participant information sheet was provided to the interviewee followed by a thorough verbal explanation of the research. Following this, participants were invited to indicate their consent to participate in the research. During the interview, enumerators followed a script and recorded the respondent’s answers directly in a tablet device using KoBo Collect. Interviewers were trained in data collection techniques, ethical conduct of research and COVID-19 preventive measures prior to initiation of the field work.

### Data analysis

Data captured in KoBo Collect were downloaded from the server and cleaned in Microsoft Excel before importing into SPSS statistical software (IBM Inc; Chicago, IL) for analysis. Data on awareness, knowledge, attitudes and practices were described using counts and proportions. Response rates to mental health questions were high with only 2–8% of responses to individual questions reported as “don’t know/no answer”. Missing WHO-5 and PHQ-9 data were imputed using standard methods for multiple imputation in SPSS [36]. Subsequently, a total raw score ranging from 0 to 25 (WHO-5) and 0–27 (PHQ-9) was calculated for each participant as the sum of responses to individual questions. WHO-5 sum of scores were then multiplied by four to give a final score ranging between 0 (lowest mental health wellbeing) and 100 (best mental health wellbeing). The mean and standard deviation for the study population was then calculated for each measure. Further, PHQ-9 scores were also categorized into 0–4 (minimal depression), 5–9 (mild), 10–14 (moderate), 15–19 (moderately severe), and 20 or greater (severe), and described as counts and proportions. Cronbach’s alpha was used to assess internal consistency of WHO-5 and PHQ-9 data, while Pearson’s correlation was used to assess the extent of agreement between WHO-5 and PHQ-9 data.

Univariable logistic regression was performed to investigate factors associated with current soap use and whether a person indicated that they agreed/strongly agreed that they had difficulty paying rent or buying food and medicine because of COVID-19. The independent variable of interest was the presence of depressive symptoms (classified as high if PHQ ≥ 15 and low if PHQ < 15). Other potential explanatory (confounding) variables included age, gender, education, and earnings. In addition, the effect of positive attitudes towards ease of hand washing and whether it was important to prevent COVID-19 were explored in the model on current soap use. Explanatory variables with a *p*-value < 0.25 in univariable logistic regression models were considered for inclusion in multivariable logistic models. After fitting the full models, variables with the highest *p*-value were removed in a stepwise fashion until the final models included only variables with a *p*-value ≤ 0.05 (backwards stepwise regression). The independent variable, depressive symptoms, was retained in all final models as the variable of interest. Overall significance and goodness of fit of final models was assessed using the Omnibus Test of model coefficients and Nagelkerke (pseudo) R Square, respectively. Two-way interactions between explanatory variables were examined during the model building process but were found to be non-significant (*p*-value > 0.05) and thus excluded from the final models. Collinearity was assessed using Pearson’s correlation coefficient and deemed present if the correlation coefficient was > 0.7 between two or more predictors.

## Results

### Demographic characteristics, living situation and livelihood strategy

The demographic characteristics, living situation and livelihood strategy of 585 respondents is shown in Table 1. The median age of respondents was 35 years (range from 18 to 79). Males made up slightly more than half (56%) of the respondents and most of them were either the household head (*n* = 261; 45%) or wife/husband of the household head (*n* = 281; 48%); remaining respondents were other family members such as son/daughter and close relatives. The mean household size was 6.4 people (standard deviation [SD] 2.6). Most respondents had low levels of literacy, with more than 40% (*n* = 238) having never attended formal schooling. 81% (*n* = 475) of the interviewees lived in temporary dwellings (“buul”; tent like shelter constructed from wood, bags, plastics, and other locally available materials). On average, dwellings comprised 1.82 (SD 0.83) rooms. More than half (*n* = 311; 53%) of the respondents stated they usually travel from home to their work place every day. Daily wages were the main source of household income in the last 6 months in the majority of households (*n *= 364; 62%) while humanitarian assistance was the main source of income in around 13% of households (*n* = 75). More than half of the respondents indicated that household earnings were < 5 USD in the previous week (*n* = 331; 57%).

**Table 1:** Demographic characteristics, living situation and livelihood strategy of respondents living in randomly selected IDP settlements in Somalia (*n* = 585)

Characteristic		Number	Percentage
*Respondent characteristics*			
Age			
18–30 years		210	35.9
31–50 years		295	50.4
51 years or older		80	13.7
Gender			
Male		327	55.9
Female		258	44.1
Relationship to head of household			
Head		261	44.6
Wife/husband		281	48.0
Other		43	7.4
Highest level of education			
Not formal schooling		238	40.7
Quranic school		194	33.2
Primary		79	13.5
Secondary or above		74	12.6
*Household characteristics*			
Camp location, n (%)			
Mogadishu		234	40.0
Hargeisa		117	20.0
Garowe		39	6.7
Baidao		195	33.3
Type of dwelling			
Buul		475	81.2
Brick		57	9.7
Other		53	9.1
Source of water for handwashing			
Water trucking or kiosk		285	48.7
Tube well		167	28.5
Deep well		88	15.0
Rain water		81	13.8
Other		71	12.1
*Livelihood*			
Main sources of household income in last 6 months ^a^			
Employment/daily wage		364	62.2
Unemployment		95	16.2
Social/humanitarian assistance		75	12.8
Other		51	8.7
Sectors of employment of household members ^a^			
Service		142	24.3
Construction		151	25.8
Public		70	12.0
Petty trade, industry and agriculture		49	8.4
Other		154	26.3
Earnings in previous week			
1.5 USD or less		162	27.7
1.5-5 USD		169	28.9
5–10 USD		139	23.8
10 USD or more		85	14.5
Don’t know/no answer		30	5.1

^a^ More than one response allowed; do not add up to 100%

### COVID-19 awareness, knowledge and attitudes

Over 89% of the respondents (*n* = 523) stated that they had heard of and/or knew about COVID-19 and most stated they knew of ways that COVID-19 could be prevented (*n* = 423; 72%) or treated (*n* = 292; 50%). Table 2 shows the perceived symptoms, transmission routes, methods of prevention and treatments of COVID-19. Most participants identified fever, cough and difficult breathing as the main symptoms, confirming good knowledge of the infection. Even though most recognized valid transmission pathways, some participants still conveyed irrelevant transmission pathways such as contact with contaminated animals or drinking unclean water.

**Table 2:** COVID-19 knowledge of respondents living in randomly selected IDP settlements in Somalia (*n* = 585)

Knowledge		Number	Percentage
Main symptoms of COVID-19 ^a^			
Fever		467	79.8
Cough		434	74.2
Shortness of breath/breathing difficulties		319	54.5
Headache		224	38.3
Muscle pain and tiredness		202	34.5
Diarrhea		26	4.4
Don’t know/no answer		10	1.7
No symptoms		5	0.9
Transmission of COVID-19 ^a^			
Being exposed to sneezes and coughs		487	83.3
Touching contaminated objects/surfaces		362	61.9
Contact with contaminated animals		135	23.1
Drinking unclean water		86	14.7
Eating contaminated food		56	9.6
Blood transfusion		23	3.9
Mosquito bites		21	3.6
Don’t know/no answer		13	2.2
Prevention of COVID-19 ^a^			
Wash your hands regularly using sanitizer or soap and water		398	68.0
Cover your mouth and nose when coughing or sneezing		367	62.7
Avoid close contact with anyone who has a fever and cough		278	47.5
Avoid social gatherings, crowded places and keeping physical distance		165	28.2
Avoid touching face and nose with my hands		163	27.9
Drink only treated water		56	9.6
Avoid unprotected direct contact with live animals and surfaces in contact with animals		26	4.4
Cook meat and eggs well		20	3.4
Sleep under the mosquito net		18	3.1
Eliminate standing water		9	1.5
Treatment ^a^			
Ginger		202	34.5
Garlic		166	28.4
Praying to Almighty		167	28.5
Honey		145	24.8
Lemon and/or vitamin C		132	22.6
Herbal medicines		113	19.3
Black seed		82	14.0
Other		5	0.9

^a^ More than one response allowed; do not add up to 100%

Eighty-one per cent (*n* = 474) of the respondents considered it important to take actions to prevent the COVID-19. Nonetheless, knowledge of appropriate preventive practices was only moderate, with regular hand washing with soap and social distancing only cited by around 70% and 50% of respondents, respectively (Table 2). Further, some participants cited irrelevant preventive measures such as avoiding contact with animals and sleeping under the mosquito net.

Participants identified a number of traditional and herbal remedies that could be used to treat COVID-19 (Table 2). However, only 197 (34%) stated that they think these remedies work; 388 (66%) were uncertain or hesitant about their effectiveness.

### Handwashing practices and attitudes

70% of respondents (*n* = 407) stated that they had changed their handwashing practices since the pandemic emerged. Of these, 362 (62%) said they washed their hands more frequently, while 253 (43%) said they washed their hands for longer than before. Nonetheless, when asked about current handwashing practices at home, handwashing practices were deemed inadequate in most participants. For instance, only 316 (55%) indicated that they currently use soap/ash while 422 (72%) indicated they currently wash their hands in a bowl of water (sharing with other people). Around one third (*n* = 183; 31%) of respondents reported that hand washing was not easy/difficult. Reasons for this included lack of soap (*n* = 447; 77%) and no water (*n* = 138; 22%).

### Perceived impact of COVID-19 restrictions on livelihoods, food security and humanitarian assistance

**Fig. 1:**
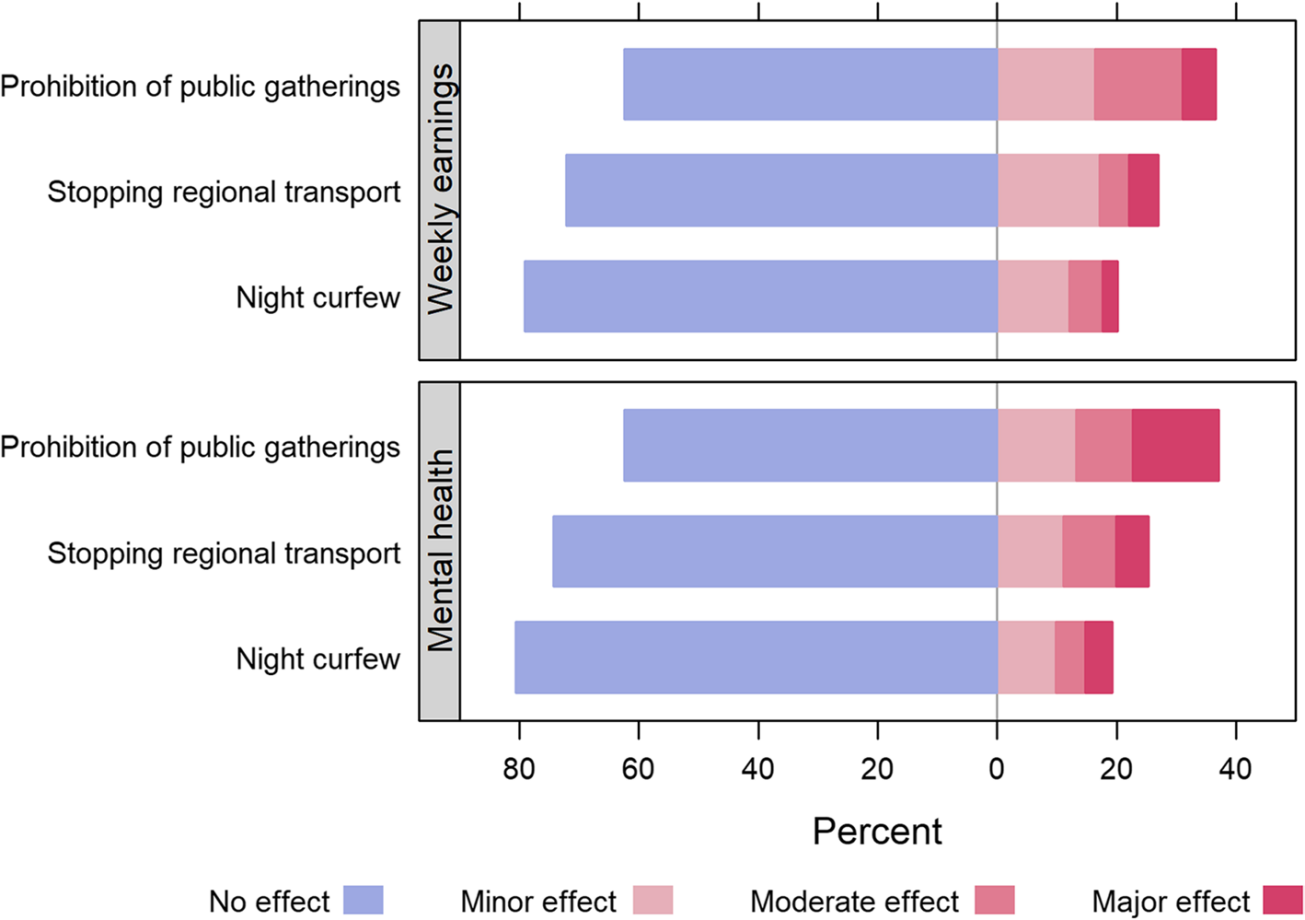
Perceived impact of COVID-19 restrictions on weekly earnings (top panel) and mental health (bottom panel) of respondents living in randomly selected IDP settlements in Somalia (*n* = 585)

The perceived impact of COVID-19 restrictions on weekly earnings is shown in Fig. 1 (top panel). The largest impact was perceived to be related to the prohibition of public gatherings, where around 20% of respondents (*n* = 119) indicated this had a moderate or major effect on their weekly earnings. Examples of such impacts included: prohibiting community meetings to solve local disputes and closure of food distribution sites where many people usually gather. Of the 311 who indicated they usually travel to work, around two thirds (*n* = 202; 65%) of respondents indicated that daily work-related travels had not changed since the introduction of government directives, while others reported minor changes such as decreased number of passengers (*n* = 105; 34%) and stopping of services or diversion to other directions (*n* = 66; 21%).

**Fig. 2:**
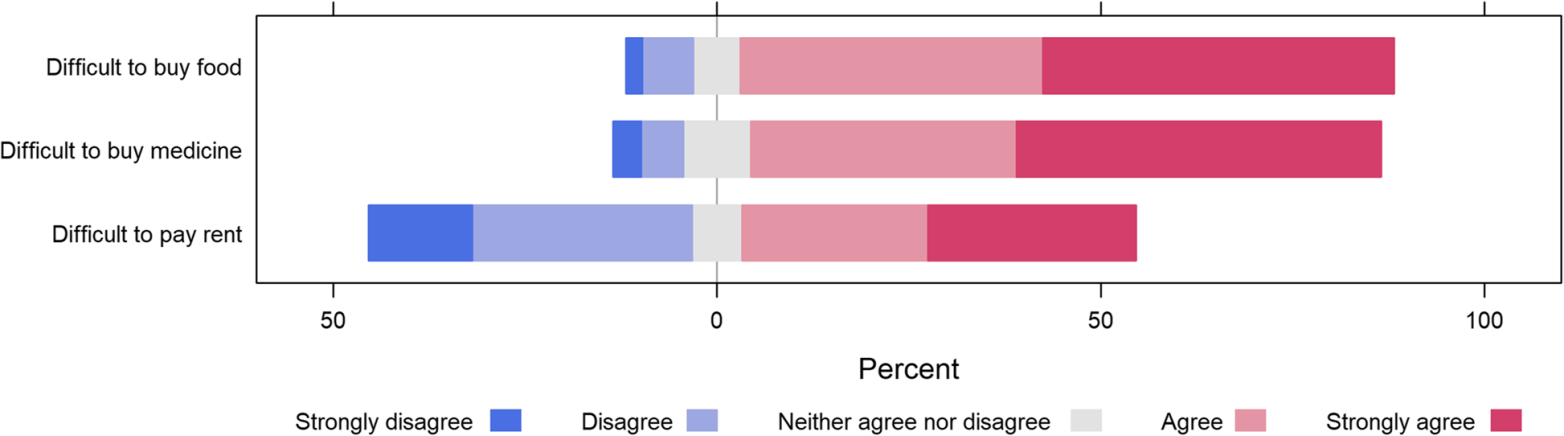
Perceived impact of COVID-19 and associated restrictions on ability to pay for rent and buy food and medicine in randomly selected IDP settlements in Somalia (*n* = 585)

Overall, a majority of respondents agreed or strongly agreed that COVID-19 and the associated restrictions had impacted their ability to pay for rent, as well as buy food and medicines (Fig. 2). A majority (*n* = 452; 77%) of respondents perceived that food prices had increased since the start of the pandemic and associated restrictions. Respondents stated that this affected various goods including: imported maize, rice and spaghetti (*n* = 385; 66%); imported agricultural products (*n* = 342; 59%); locally produced agricultural produce (*n* = 321; 55%); and other food items (*n* = 16; 3%).

Around two thirds (*n* = 383; 66%) of participants said humanitarian aid from NGOs had been reduced since the start of the pandemic. According to participants this included reductions in food distribution (*n* = 240; 58%), cash transfer (*n* = 310; 53%), nutrition for children (*n* = 260; 44%) and health services (*n* = 200; 34%). 42% of respondents (*n* = 244) reported that this contributed to complete loss of income while 20% (*n* = 119) stated this led to partial loss of income; very few people (*n* = 12; 2.5%) mentioned that they had started alternative income generation due to the reduced humanitarian assistance.

### Mental health and wellbeing

The mean WHO-5 score for the study population was 44.2 out of 100 (SD 24.4). 70% of participants had a raw score of ≤ 52, suggesting the majority were potentially experiencing clinical depression. The mean PHQ-9 score out of 27 was 18.6 (SD 5.2). Table 3 shows the frequency of depressive symptoms by severity category; nearly 80% of respondents scored in a range indicating they had severe or moderately severe depressive symptoms. Around two thirds (*n* = 357; 68%) of respondents indicated that these problems made it very or extremely difficult to do their work, take care of things at home, and/or get along with people. Out of 458 respondents with severe or moderately severe depressive symptoms, 393 (86%) were young or middle-aged people, and 262 (57%) were female (Table 3). Both WHO-5 and PHQ-9 had strong internal consistency (α = 0.82 and 0.83, respectively) and there was strong negative correlation between WHO-5 and PHQ-9 responses (*r* = -0.3; *p*-value < 0.001).

When asked whether they felt better, the same or worse than before COVID-19, 402 (74%) respondents indicated they felt worse than before. When asked if how various government restrictions affected how participants were feeling lately, the prohibition on public gatherings was again cited as most important (Fig. 1, bottom panel); 112 (23%) respondents stated this had a moderate or major impact on how they were feeling. In contrast, the night curfew and stopping of regional transport had no or minimal effect on how respondents were feeling (*n* = 501 and 485; 89% and 83%, respectively).

**Table 3:** Frequency of depressive symptoms in respondents living in randomly selected IDP settlements in Somalia (*n* = 585) stratified by age category and gender, as assessed using the Patient Health Questionnaire-9 (PHQ-9). Frequency counts are based on pooled estimate for multiple imputations, rounded to nearest whole number

PHQ-9 Category	Raw PHQ-9 score (minimum –maximum)	Percent (%) of respondents with depressive symptoms					
		Overall	By age category ^a^			By gender ^b^	
			Young age(18–30 years)	Middle-aged(31–50 years)	Older age(≥ 51 years)	Female	Male
None	0–4	2 (0.3)	1 (0.5)	1 (0.3)	0 (0)	1 (0.3)	1 (0.4)
Mild	5–9	28 (4.8)	8 (4)	15 (5)	5 (6.3)	15 (4.5)	14 (5.3)
Moderate	10–14	97 (16.5)	33 (15.8)	53 (17.8)	11 (13.8)	49 (15)	48 (18.5)
Moderately severe	15–19	196 (33.4)	85 (40.6)	84 (28.6)	26 (32.5)	107 (32.5)	89 (34.5)
Severe	20–27	262 (44.9)	82 (39.1)	142 (48.2)	38 (47.5)	156 (47.7)	107 (41.3)

^a^ Pearson chi-square: *p* > 0.1 for all imputations

^b^ Pearson chi-square: *p* > 0.1 for all imputations

### Multivariable regression analysis

Final results of multivariable logistic regression investigating factors associated with current use of soap and difficulty paying rent, buying food or medicine due to COVID-19 is shown in Table 4. (Full results of univariable logistic regression are shown in Table S1). Adjusted for other factors, high depressive symptoms were significantly associated with lower frequency of current soap use, and higher frequency of agreement that it was difficult to buy food due to COVID-19. Specifically, adjusted for earnings and knowledge that handwashing prevents COVID-19, respondents with high depressive symptoms were 70% less likely to say they were using soap currently compared to people with low depressive symptoms (48.9% vs. 73.6%; adjusted odds ratio [aOR] = 0.3; 95% confidence interval [CI] = 0.2, 0.7; *P* < 0.001). In contrast, adjusted for earnings, respondents with high depressive symptoms were more than twice as likely to say they agreed or strongly agreed that they had difficulty buying food because of COVID-19 compared to people with low depressive symptoms (87.0% vs. 78.3%; aOR = 2.2; 95% confidence interval [CI] = 1.1, 4.3; *P* = 0.02). Depressive symptoms were not associated with difficulty paying rent or buying medicine after adjusting for other factors.

**Table 4:** Results of multivariable logistic regression investigating factors associated with current use of soap and difficulty paying rent, buying food or medicine due to COVID-19 amongst respondents living in randomly selected IDP settlements in Somalia. The independent variable, depressive symptoms, was retained in all models as the variable of interest

Independent variable	Frequency distribution of dependent variable		B	SE	aOR (95% CI)	*P*-value
	Yes, n (%)	No, n (%)				
*Dependent variable: Currently uses soap* ^a^						
Depressive symptoms ^b^						
Low (PHQ-9 < 15)	92 (72.6)	35 (27.4)			1	
High (PHQ-9 ≥ 15)	224 (48.9)	234 (51.1)	-1.1	0.4	0.3 (0.2, 0.7)	< 0.001
Earnings in previous week						
1.5 USD or less	29 (17.9)	133 (82.1)			1	
1.5-5 USD	85 (50.3)	84 (49.7)	1.6	0.3	5.1 (2.8, 9.4)	< 0.001
5–10 USD	117 (84.2)	22 (15.8)	3.3	0.4	26.1 (12.3, 55.5)	< 0.001
10 USD or above	71 (83.5)	14 (16.5)	3.5	0.5	31.6 (12.5, 80.1)	< 0.001
Knowledge that hand washing prevents COVID-19						
No	5 (20.0)	20 (80.0)	3.0	0.8	1	
Yes	219 (55.0)	179 (45.0)			20.5 (4.1, 103.4)	< 0.001
*Dependent variable: Difficulty paying rent due to COVID-19* ^c^						
Depressive symptoms ^b^						
Low (PHQ-9 < 15)	70 (62.8)	41 (37.2)			1	
High (PHQ-9 ≥ 15)	174 (47.9)	190 (52.1)	-0.4	0.3	0.7 (0.4, 1.2)	0.16
Earnings in previous week						
1.5 USD or less	18 (13.8)	112 (86.2)			1	
1.5-5 USD	96 (64.9)	52 (35.1)	2.4	0.3	11.2 (6.1, 20.5)	< 0.001
5–10 USD	86 (69.9)	37 (30.1)	2.6	0.3	13.7 (7.3, 25.9)	< 0.001
10 USD or above	41 (59.4)	28 (40.6)	2.2	0.4	8.7 (4.4, 17.5)	< 0.001
*Dependent variable: Difficulty buying food due to COVID-19* ^d^						
Depressive symptoms ^b^						
Low (PHQ-9 < 15)	97 (78.3)	27 (21.7)			1	
High (PHQ-9 ≥ 15)	389 (87.0)	58 (13.0)	0.8	0.3	2.2 (1.1, 4.3)	0.02
Earnings in previous week						
1.5 USD or less	146 (91.3)	14 (8.8)			1	
1.5-5 USD	116 (69.0)	52 (31.0)	-1.5	0.3	0.2 (0.1, 0.4)	< 0.001
5–10 USD	121 (91.7)	11 (8.3)	0.2	0.4	1.2 (0.5, 2.9)	0.63
10 USD or above	78 (92.9)	6 (7.1)	0.3	0.5	1.4 (0.5, 3.8)	0.51
*Dependent variable: Difficulty buying medicine due to COVID-19* ^e^						
Depressive symptoms ^b^						
Low (PHQ-9 < 15)	100 (81.2)	23 (18.8)			1	
High (PHQ-9 ≥ 15)	350 (82.4)	75 (17.6)	0.0	0.3	1.0 (0.5, 2.0)	0.95
Education						
No formal schooling	180 (83.3)	36 (16.7)			1	
Quranic school	134 (73.6)	48 (26.4)	-0.6	0.3	0.5 (0.3, 0.9)	0.02
Primary	68 (88.3)	9 (11.7)	0.5	0.4	1.7 (0.7, 4.0)	0.22
Secondary or above	68 (93.2)	5 (6.8)	0.7	0.5	2.0 (0.7, 4.0)	0.18
Earnings in previous week						
1.5 USD or less	132 (91.0)	13 (9.0)			1	
1.5-5 USD	103 (66.5)	52 (33.5)	-1.7	0.3	0.2 (0.1, 0.3)	< 0.011
5–10 USD	116 (86.6)	18 (13.4)	-0.7	0.4	0.5 (0.2, 1.1)	0.09
10 USD or above	78 (91.8)	7 (8.2)	-0.2	0.5	0.9 (0.3, 2.0)	0.76

*SE* Standard error, *aOR* adjusted odds ratio, *CI* Confidence interval, *PHQ-9* Patient Health Questionnaire-9

^a^ Final model based on complete data for *n* = 398 people. Omnibus test of model coefficients: *p* < 0.001 for all imputations. Nagelkerke R square ranged between 0.45 and 0.46 for model based on each imputation

^b^ Pooled estimate for multiple imputations, rounded to nearest whole number

^c^ Final model based on complete data for *n* = 385 people. Omnibus test of model coefficients: *p* < 0.001 for all imputations. Nagelkerke R square ranged between 0.28 and 0.29 for model based on each imputation

^d^ Final model based on complete data for *n* = 448 people. Omnibus test of model coefficients: *p* < 0.001 for all imputations. Nagelkerke R square ranged between 0.14 and 0.17 for model based on each imputation

^e^ Final model based on complete data for *n* = 426 people. Omnibus test of model coefficients: *p* < 0.001 for all imputations. Nagelkerke R square 0.16 for models based on all imputations

## Discussion

Close to 19% of Somalia’s 16 million population are internally displaced and live in informal settlements where they are vulnerable to disasters and have very low resilience capacity. Humanitarian and other interventions that are grounded in evidence-based findings are needed to support these populations yet priorities and experiences of IDPs are often understudied. This study is unique for revealing the interrelationship between COVID-19 restrictions, livelihood, hand hygiene practicality and mental health and wellbeing in Somalia’s conflict-affected, displaced communities. Although knowledge and awareness of COVID-19 was relatively good at the time of the survey, practices such as regular hand washing with soap were not being practiced by 45% of the respondents largely due to difficulties accessing soap and water. Further, livelihoods and food security of these internally displaced communities were impacted in two ways. Firstly, social distancing directives aimed at restricting public gatherings negatively impacted daily earnings by limiting access to markets and led to the reduction of humanitarian food distribution at crowding points. Secondly, a sharp price increase in basic food items impacted people’s ability to buy these items. This study is the first to show that existing high levels of depression in Somalia’s internally-displaced communities were potentially compounded by COVID-19 restrictions such as prohibiting public gatherings. Further, it showed that people with severe or moderately severe depression (PHQ-9 ≥ 15) were less likely to practice good hand hygiene practices and more likely to cite difficulties buying food because of COVID-19.

COVID-19 overwhelmed the health systems of countries that scored well in the 2019 pandemic preparedness assessment [12]. In contrast, it was reported early in the pandemic that COVID-19 had a comparatively lower impact on less prepared African countries which reported lower cases, potentially due to younger populations, delayed pandemic spread, decisive response and other debated explanations [37]. Nevertheless, there was a significant socio-economic impact on the livelihoods of African communities that depend on daily earning wages [38], including in Somalia [39]. Livelihood concerns expressed by participants in this study are similar to those reported in recent studies conducted in Somalia’s conflict-affected and displaced communities that similarly reported livelihood interruptions, a decrease in household income, and failure to buy basic food items due to movement restrictions [40, 41]. In this study, earnings in previous week had an inconsistent association with difficulty paying rent, buying food and/or medicine. Counter-intuitively, people with higher earnings in previous week were more likely to say they had difficulty paying rent due to COVID-19. We speculate that people with higher income may be accustomed to being more self-reliant and thus may have perceived greater difficulty during the pandemic. This highlights the intrinsic vulnerability of the population to multiple shocks and points to the need for future pandemic prevention strategies to specifically address displaced communities as they cannot observe movement restrictions that hobble their daily earnings for survival. Other researchers have recognized that such interventions duplicated international responses without being contextualized to local circumstances [41].

We report a relatively good knowledge of the pandemic by displaced communities in Somalia which corresponds well with other surveys conducted by humanitarian agencies [42, 43]. This is likely due to radio campaigns and distribution of translated posters by local governments and NGOs, aimed at improving awareness of COVID-19 and its prevention. Nonetheless, on the basis of their description of current practices, hand washing was deemed to be inadequate in nearly half of the respondents. Poor hand hygiene can be attributed in part to the general lack of adequate clean water; in 2022, Somalia’s Water, Sanitation, and Hygiene (WASH) cluster reports inadequate clean water for appropriate sanitation for more than 1.4 million displaced communities [44].

COVID-19 is associated with a high prevalence of depression and anxiety [45] which can be more precarious in low resource settings [46] such as conflict-affected communities in sub-Saharan countries that struggle to access primary health care [47]. Forcibly displaced children and youth are, in particular, considered amongst the most vulnerable to mental health impacts during the COVID-19 pandemic [48]. We report a high level of depressive symptoms among Somalia’s displaced communities and some evidence that mental health and wellbeing likely deteriorated further due to the pandemic and associated restrictions. Somalia has a relatively young population (median age is 16.7 years [49]) which likely explains why young and middle-aged people were over-represented amongst people with severe or moderately severe depressive symptoms; in fact levels of moderate or severe depression were relatively similar across all age groups in this study. This contrasts with a report of higher depression rates among older aged Rohinga refugees communities in Bangladesh during the pandemic [50]. The finding that females were slightly over-represented amongst people with moderate or severe depression is consistent with global trends of depression in the community [51]. Mental health and wellbeing has been neglected for decades in Somalia due to the prolonged conflict and lack of health care services [52]. This mental health burden should be a priority concern for policymakers and humanitarian agencies, particularly given potential negative knock-on impacts in terms of hand hygiene practice and ability to buy food. Similar mental health impacts of lockdowns have been reported in other conflict-affected countries such as Syria, where reduced ability to earn and provide food were all highlighted as the main concerns [53]. In recent years, the WHO has developed scalable interventions to support mental health including the Self-Help Plus (SH+) intervention that has been shown to reduce distress, prevent mental disorders and enhance wellbeing in refugee populations [54–56]. The results of the current study highlight the need to explore the opportunities that may exist for implementing scalable interventions such as SH + in Somalia. The findings also highlight the need to coordinate support at times of crises across different levels of displaced people’s social environment [57].

Importantly, we found evidence of inadequate hand hygiene practice particularly amongst people with high depressive symptomatology. This mirrors findings from a longitudinal study in China which reported a positive correlation between good hand hygiene and better mental health during the pandemic [58]. Similarly, a study in three South American countries revealed a relationship between poor mental health and inadequate handwashing in ordinary (pre-pandemic) times [59]. We did not explore possible mechanisms for this association in our study, however we speculate that a sense of apathy and/or despondency may mean people with depression are less likely to use soap and/or wash their hands. Indeed, people with depressive symptoms in this study were less likely to indicate that handwashing was difficult than those without such symptoms (data not shown). Alternatively, attitudes towards the importance of handwashing with soap may vary between people with and without depression. In Malawi, for example, vulnerable populations with poor mental health believed handwashing with soap is expensive and thus tended not to practice regular handwashing [60].

This study has a number of weaknesses and strengths which should be mentioned. We recruited only internally displaced people in the selected camps which limits our understanding of how the experiences of these people compare to the ways in which members of the host communities and refugees from other countries (like Yemen and Ethiopia) are coping with the pandemic. Nonetheless, the use of a sampling framework with random selection does mean that the findings are likely representative of and can be generalized to the wider IDP population in Somalia. Being a cross-sectional study, we cannot comment on how the impacts of COVID-19 evolved over the pandemic period, although the timing of data collection (December 2020 and March 2021) did coincide with a period of widespread transmission of COVID-19 in Somalia and thus we were able to document the impacts of the pandemic as they were occurring at that time. We did not ask participants if they had personal experience with COVID-19 (self or household members). This would have provided greater insight into household-level impacts of the pandemic, which may have varied between directly and indirectly affected respondents. Lastly, even though the mental health tools used in this study have been previously deployed to internally displaced people [34, 61], there was no rigorous adaptation of the instruments to the Somali setting. Future research in Somalia would benefit from producing a context specific version of both WHO-5 and PHQ-9 tools.

## Conclusion

In conclusion, we report significant impacts on livelihood, food security and mental health and wellbeing of Somalia’s internally displaced during the COVID-19 pandemic. Whilst campaigns to improve awareness led to high levels of knowledge of the pandemic, preventive measures such as handwashing were difficult to implement in this setting. Further, restrictions such as the ban on public gatherings limited people’s ability to earn wages and decreased their access to humanitarian aid. Consequently, people had difficulties buying food, medicine and/or paying for rent. A high burden of depressive symptomatology was also documented and associated with lower frequency of soap use and reported difficulties buying food. Uninterrupted livelihood support and mental health services are desperately needed in Somalia’s IDP camps during the recovery period. Sustaining access to such social support should be a priority in future pandemic planning.

## Supplementary Information

**Additional file 1: Table S1.** Results of univariable investigating factors associated with currently using soap and difficulty paying rent, buying food or medicine due to COVID amongst respondents living in randomly selected IDP settlements in Somalia. Response rates to individual questions are indicated. Where response rates are less than 100% (i.e. 585) respondents indicated the question was “not applicable”. 

## Data Availability

The datasets used and/or analysed during the current study are available from the corresponding author on reasonable request.

## Abbreviations

aOR: Adjusted odds ratio
CCM: Camp Coordination and Camp Management
CI: Confidence interval
IDP: Internally displaced person
IOM: International Organization of Migration
PHQ-9: Patient Health Questionnaire-9
WHO-5: 5-item World Health Organization Wellbeing Index
