# Supplementary material for: COVID-19 and its prevention in internally displaced person (IDP) camps in Somalia: impact on livelihood, food security and mental health
Source: BMC Public Health. 2022 Dec 22;22:2414. doi: 10.1186/s12889-022-14878-z (PMC9779940; doi:10.1186/s12889-022-14878-z)
Supplement: Supplementary file 1 — Additional file 1: Table S1. Results of univariable investigating factors associated with currently using soap and difficulty paying rent, buying food or medicine due to COVID amongst respondents living in randomly selected IDP settlements in Somalia. Response rates to individual questions are indicated. Where response rates are less than 100% (i.e. 585) respondents indicated the question was “not applicable”. [file 12889_2022_14878_MOESM1_ESM.docx]

**Table S1.** Results of univariable investigating factors associated with currently using soap and difficulty paying rent, buying food or medicine due to COVID amongst respondents living in randomly selected IDP settlements in Somalia. Response rates to individual questions are indicated. Where response rates are less than 100% (i.e. 585) respondents indicated the question was “not applicable”.

| Characteristic | Currently uses soap  n=585 | | | Difficulty paying rent  n=475 | | | Difficulty buying food  n=571 | | | Difficulty buying medicine  n=548 | | |
| --- | --- | --- | --- | --- | --- | --- | --- | --- | --- | --- | --- | --- |
|  | Yes, n (%) | No, n (%) | P-value | Yes, n (%) | No, n (%) | P-value | Yes, n (%) | No, n (%) | P-value | Yes, n (%) | No, n (%) | P-value |
| Age | | | | | | | | | | | | |
| 18 – 30 years | 133 (63.3) | 77 (36.7) | - | 104 (63.4) | 60 (36.6) |  | 170 (82.5) | 36 (17.5) |  | 157 (78.5) | 43 (21.5) |  |
| 31 – 50 years | 138 (46.8) | 157 (53.2) | <0.001 | 103 (43.1) | 136 (56.9) | <0.001 | 252 (87.8) | 35 (12.2) | 0.10 | 230 (84.9) | 41 (15.1) | 0.08 |
| 51 years or older | 45 (56.3) | 35 (43.8) | 0.27 | 37 (51.4) | 35 (48.6) | 0.08 | 64 (82.1) | 14 (17.9) | 0.93 | 63 (81.8) | 14 (18.2) | 0.54 |
| Gender | | | | | | | | | | | | |
| Male | 137 (53.1) | 121 (46.9) | - | 108 (53.7) | 93 (46.3) |  | 213 (85.9) | 35 (14.1) |  | 192 (79.7) | 49 (20.3) |  |
| Female | 179 (54.7) | 148 (45.3) | 0.69 | 136 (49.6) | 138 (50.4) | 0.38 | 273 (84.5) | 50 (15.5) | 0.65 | 258 (84.0) | 49 (16.0) | 0.19 |
| Education | | | | | | | | | | | | |
| No formal schooling | 123 (51.7) | 115 (48.3) | - | 92 (51.1) | 88 (48.9) |  | 202 (86.3) | 32 (13.7) |  | 180 (83.3) | 36 (16.7) |  |
| Quranic school | 98 (50.5) | 96 (49.5) | 0.81 | 88 (50.9) | 85 (49.1) | 0.96 | 152 (79.6) | 39 (20.4) | 0.07 | 134 (73.6) | 48 (26.4) | 0.02 |
| Primary | 49 (62.0) | 30 (38.0) | 0.11 | 39 (60.0) | 26 (40.0) | 0.22 | 67 (90.5) | 7 (9.5) | 0.34 | 68 (88.3) | 9 (11.7) | 0.30 |
| Secondary or above | 46 (62.2) | 28 (37.8) | 0.12 | 25 (43.9) | 32 (56.1) | 0.34 | 65 (90.3) | 7 (9.7 | 0.38 | 68 (93.2) | 5 (6.8) | 0.04 |
| Earnings in previous week | | | | | | | | | | | | |
| 1.5 USD or less | 29 (17.9) | 133 (82.1) | - | 18 (13.8) | 112 (86.2) | - | 146 (91.3) | 14 (8.8) | - | 132 (91.0) | 13 (9.0) | - |
| 1.5-5 USD | 85 (50.3) | 84 (49.7) | <0.001 | 96 (64.9) | 52 (35.1) | <0.001 | 116 (69.0) | 52 (31.0) | <0.001 | 103 (66.5) | 52 (33.5) | <0.001 |
| 5-10 USD | 117 (84.2) | 22 (15.8) | <0.001 | 86 (69.9) | 37 (30.1) | <0.001 | 121 (91.7) | 11 (8.3) | 0.90 | 116 (86.6) | 18 (13.4) | 0.24 |
| 10 USD or above | 71 (83.5) | 14 (16.5) | <0.001 | 41 (59.4) | 28 (40.6) | <0.001 | 78 (92.9) | 6 (7.1) | 0.66 | 78 (91.8) | 7 (8.2) | 0.85 |
| Don’t know/prefer not to say | 14 (46.7) | 16 (53.3) | - | 2 (40.0) | 3 (60.0) |  | 25 (92.6) | 2 (7.4) | - | 98 (82.4) | 21 (17.6) | - |
| Depressive symptoms ^a^ | | | | | | | | | | | | |
| Low (PHQ-9 <15) | 92  (72.6) | 35  (27.4) | - | 70  (62.8) | 41  (37.2) | - | 97  (78.3) | 27  (21.7) | - | 100  (81.2) | 23.2 (18.8) | - |
| High (PHQ-9 ≥15) | 224  (48.9) | 234  (51.1) | <0.001 | 174  (47.9) | 190  (52.1) | 0.01 | 389  (87.0) | 58  (13.0) | 0.05 | 350  (82.4) | 74.8 (17.6) | 0.78 |
| Knowledge that hand-washing prevents COVID-19 | | | | | | | | | | | | |
| No | 5 (20.0) | 20 (80.0) | - | - | - | - | - | - | - | - | - | - |
| Yes | 219 (55.0) | 179 (45.0) | <0.001 | - | - | - | - | - | - | - | - | - |
| Don’t know/skipped | 92 (56.8) | 70 (43.2) | - | - | - | - | - | - | - | - | - | - |
| Attitudes towards handwashing | | | | | | | | | | | | |
| Difficult | 128 (69.9) | 55 (30.1) | - | - | - | - | - | - | - | - | - | - |
| Easy/so-so | 186 (47.8) | 203 (52.2) | <0.001 | - | - | - | - | - | - | - | - | - |
| Don’t know | 2 (15.4) | 11 (84.6) | - | - | - | - | - | - | - | - | - | - |
| Attitude towards prevention of COVID-19 | | | | | | | | | | | | |
| Not important | 32 (84.2) | 6 (15.8) | - | - | - | - | - | - | - | - | - | - |
| Important | 263 (55.5) | 211 (44.5) | <0.001 | - | - | - | - | - | - | - | - | - |
| Don’t know/skipped | 21 (28.8) | 52 (71.2) | - | - | - | - | - | - | - | - | - | - |
| ^a^  Pooled estimate for multiple imputations, rounded to nearest whole number. | | | | | | | | | | | | |
